# Sociocultural Adjustment and Well-being Among Third Culture Kids and Their Families: Protocol for a Longitudinal Study

**DOI:** 10.2196/30088

**Published:** 2022-07-01

**Authors:** Yoon Phaik Ooi, Marnie Reed, Emma Marchal-Jones, Andrea Hans Meyer, Jens Gaab

**Affiliations:** 1 Division of Clinical Psychology and Psychotherapy Department of Psychology University of Basel Basel Switzerland; 2 Department of Developmental Psychiatry Institute of Mental Health Singapore Singapore

**Keywords:** family functioning, resilience, sociocultural adjustment, stress, third culture kids, well-being

## Abstract

**Background:**

Increasing globalization has led to more families with children being relocated each year, highlighting the importance of issues, such as adjustment and psychological well-being, in this population. These children, commonly known as third culture kids, often spend a significant part of their developmental years in countries and cultures foreign to them.

**Objective:**

Our longitudinal study aims to examine the roles of cognitive, psychological, sociocultural, and family factors in the longitudinal trajectories of the well-being and sociocultural adjustment of third culture kids and their families over time.

**Methods:**

This study adopts both quantitative and qualitative procedures. Data from both procedures will be collected at baseline and at a 1-year follow-up. We aim to recruit 150 to 200 participants between 7 and 17 years old and one of their primary caregivers. After providing informed consent, participants will complete an online survey. Outcome measures include validated questionnaires on well-being and sociocultural adjustment. Predictor measures include validated questionnaires on negative self-thoughts, emotion regulation, resilience, psychological attributes, self-esteem, stress, acculturative stress, cultural intelligence, couple satisfaction, and family functioning. A multiple regression model will be used to analyze quantitative data. In addition, 15 to 20 families who participate in the online survey will be randomly selected to take part in a family interview focusing on questions related to well-being, relocation experiences, cultural issues, and challenges. A concurrent triangulation mixed methods design will be used to analyze and interpret data from both quantitative and qualitative methods.

**Results:**

As of March 15, 2022, a total of 138 children and 126 parents have completed the baseline online survey. In addition, 44 children and 48 parents have completed the 1-year follow-up online survey. A total of 8 families have completed the baseline family interview, while 4 families have completed the 1-year follow-up interview. Data analyses, transcription of the interview, and preparation for publication are on-going.

**Conclusions:**

Findings from this study would enable us to understand the adjustment processes, and risk and protective factors associated with the well-being and sociocultural adjustment of third culture kids and their families in Switzerland, which could have implications on the development of intervention programs for individuals and families to address acculturation and adjustment issues.

**International Registered Report Identifier (IRRID):**

RR1-10.2196/30088

## Introduction

Increased globalization and the expansion of multinational corporations across nations have made international assignments more common in recent years. A recent survey showed that 73% of expatriates, referred to as individuals who relocate to a new host country for a job and have a limited time frame on their assignments [1], were accompanied by a partner/spouse and 52% of expatriates had children with them [2]. Children who relocate with their expatriate parents often spend a large part of their growing up years in countries or cultures other than those of their parents [3], and are commonly known as third culture kids. Frequently, third culture kids are confronted with new challenges, such as establishing self-identity, finding a sense of belonging, adapting to a new school, and making new friends [4]. However, by living in different cultures, third culture kids have the opportunity to develop skills to manage and adapt to changes, and to experience and be more open to different cultures [5]. The ability to successfully adjust to this new environment can enrich third culture kids’ lives [6], while failure to manage these challenges can result in negative consequences for psychological well-being [7].

Unfortunately, up to 70% of relocations fail due to family reasons, such as the inability or unwillingness of family members to adapt to the foreign environment [8-10], resulting in high costs for organizations, ranging from US $250,000 to US $1 million, depending on various factors [11]. Failure of relocation is often defined as the premature return of an expatriate [8]. Research suggests that the key to successful international assignments of expatriates is understanding factors that influence their family’s well-being and sociocultural adjustment processes [11,12]. Well-being is defined as the subjective emotional state associated with positive feelings and an overall satisfaction with one’s life [13]. Studies have shown that relocation can negatively impact one’s subjective well-being, such as causing emotional and social problems, and in more severe cases, it could lead to psychological disorders, such as depression and eating disorders [14]. On the other hand, sociocultural adjustment refers to the individual’s ability to cope with daily problems in the new environment, the degree of fit and familiarity to the new host culture, and the redefinition of personal and social roles within the host environment [4,15].

Previous studies have identified various factors that promote well-being and sociocultural adjustment of third culture kids and their families. Studies involving only third culture kids found that secure attachment, open mindedness, emotional stability, and a high level of social initiative were associated with better adjustment [16]. In addition, family characteristics, such as family adaptability, family cohesion, and family communication, have been found to predict higher levels of sociocultural adjustment, with family cohesion being the strongest influence of both sociocultural adjustment and quality of life of third culture kids [16].

Similarly, studies that examined expatriate families as a whole and involved all family members as informants found that a supportive climate, good family communication, and positive feelings about the international assignment were associated with better adjustment [17]. Healthy relationships between spouses/partners were also found to be important for successful adjustment among expatriate families [9].

A 2018 review by Sterle et al [4] revealed several limitations within the current literature. First, most studies consist of cross-sectional surveys, case studies, or narratives to describe third culture kids’ well-being and adjustment. Findings from such studies do not examine well-being and sociocultural adjustment as a long-term process and do not provide any directional conclusions of the relationships between the various variables under study [4]. Second, there are limited studies that combine both quantitative and qualitative approaches, as well as the multi-informant approach. While both quantitative and qualitative approaches offer interesting findings, the use of mixed methods may enhance the credibility and confirmability of findings related to third culture kids’ well-being and adjustment [18]. On the other hand, the multi-informant approach may provide a more detailed picture of third culture kids’ functioning, which has important implications for subsequent interventions [19,20]. Finally, there are limited longitudinal studies that focus on the family as a unit and all family members. As research has indicated that the success of an expatriate assignment is dependent on the family, there is a need to examine the well-being and adjustment of all family members. As such, this study aims to address the limitations of the existing literature by using a longitudinal design that combines both quantitative and qualitative approaches. In addition, we will adopt a multi-informant approach by recruiting a child and a parent in the quantitative study and all family members in the qualitative study. We will also include a variety of measures that examine psychological, cultural, and family factors.

The term expatriate is often defined as a person who is relocated to another country by a multinational company for a temporary work assignment [21]. However, the terms expatriates and migrants are sometimes used interchangeably to refer to people who move outside their home countries for work [22]. Berry and Bell [21] highlighted several differences between expatriates and migrants in terms of their occupation, skills, compensation, etc. For instance, expatriates are often characterized as workers at executive, managerial, or professional positions, who have high skills, high compensation, and organizational benefits, while migrants are often characterized as skilled or unskilled laborers, who have low wages and no organizational benefits [21,23]. Based on these differences in definitions, we chose to use the term expatriates to define our study population. The focus on expatriates is largely driven by our study team’s clinical expertise and interests. In the last 5 years, our psychotherapy center has seen an increase in requests for psychological services among expatriate families. However, there is still a lack of evidence on how relocation and sociocultural adjustment impact the onset and maintenance of psychological disorders, as well as limited evidence-based interventions to support this population [4]. In addition, Switzerland, with an estimate of 25% foreign nationals, has been consistently rated as one of the most desirable destinations for expatriates due to its high quality of life and salaries, but at the same time, it has also been rated as one of the most difficult places to settle into [24], highlighting potential adjustment difficulties. Therefore, understanding factors that influence well-being and adjustment among expatriate families in Switzerland is an important and relevant topic.

Our first research question examines the roles of emotion regulation, self-esteem, resilience, and negative self-thoughts for well-being and adjustment among third culture kids over time. Previous research has demonstrated that emotion regulation [25,26], negative self-thoughts [27-29], resilience [30,31], and stress [32] are important psychological processes for healthy development, such as establishing relationships and adjusting to stressful situations [33], in nonthird culture kid populations. Hence, we expect that better emotion regulation skills, self-esteem, and resilience; fewer negative cognitions; and lower stress will enhance third culture kids’ well-being and sociocultural adjustment. The first hypothesis (hypothesis 1) is as follows: Increased levels of emotion regulation, self-esteem, and resilience, and decreased levels of negative self-thoughts and stress can significantly predict increased levels of well-being and adjustment among third culture kids over time.

Our second research question examines the roles of cultural intelligence and acculturative stress on well-being and adjustment among third culture kids over time. Cultural intelligence is defined as “an individual’s capability to function and manage effectively in culturally diverse settings” [34], and has been widely explored as a factor of success in the relocation of adult expatriates [35-37] and in the adjustment and well-being of migrant populations [38]. On the other hand, acculturative stress (eg, difficulties assimilating to the beliefs, values, and norms of a host culture) has been found to be a strong predictor of depressive symptoms and suicidal ideation among migrant families [39]. As such, we expect to find that higher cultural intelligence and lower acculturative stress are associated with higher levels of well-being and sociocultural adjustment among third culture kids. The second hypothesis (hypothesis 2) is as follows: Increased levels of cultural intelligence and decreased levels of acculturative stress significantly predict increased levels of well-being and adjustment among third culture kids over time.

Our third research question examines the roles of family functioning, couple satisfaction, and parental stress in well-being and adjustment among third culture kids over time. As the place of residence remains unstable, the family constitutes the stable part in a third culture kid’s life during relocation. Thus, the family serves as an important source of social and emotional support during the relocation experience [4,40]. Previous research has demonstrated the impact of family functioning, parental stress, and couple satisfaction on child well-being [41-45]. Hence, we expect to find higher levels of family functioning and couple satisfaction, and lower levels of parental stress to be associated with higher levels of well-being and sociocultural adjustment among third culture kids. The third hypothesis (hypothesis 3) is as follows: Increased levels of family functioning and couple satisfaction, and decreased levels of parental stress significantly predict increased levels of well-being and adjustment among third culture kids over time.

Our fourth research question aims to obtain a detailed understanding of the transition process and adjustment to Switzerland from a family perspective. In addition, it aims to further understand the factors that influence sociocultural adjustment and well-being.

## Methods

### Study Design

This is a longitudinal study that adopts both quantitative and qualitative procedures. Data from both procedures will be collected at baseline and at a 1-year follow-up. To answer research questions 1 to 3, a quantitative procedure using an online questionnaire will be used to obtain information from the participants. To answer research question 4 on the process of transitioning and adjustment in Switzerland, a concurrent triangulation mixed methods design will be used. This design includes concurrent but separate data collection from both quantitative and qualitative procedures, data analysis, and data interpretation [18]. Equal priority will be given to data obtained from both qualitative and quantitative procedures.

### Participants and Study Procedures

We aim to recruit 150 to 200 child/adolescent participants and one of their parents. Potential participants will be contacted through various sources, such as social media, expat associations, international schools, and multinational companies. The inclusion criteria are as follows: age between 7 and 17 years for children, employment of a parent being the primary reason for the family’s relocation to Switzerland, medium to high household income levels (greater than CHF 100,000 [US $100,000]), and ability to understand and speak English. As highlighted in the literature [21], the inclusion criterion of a high income level in our study is meant to include those defined as expatriates and exclude those characterized as laborers and domestic workers.

**Figure 1:**
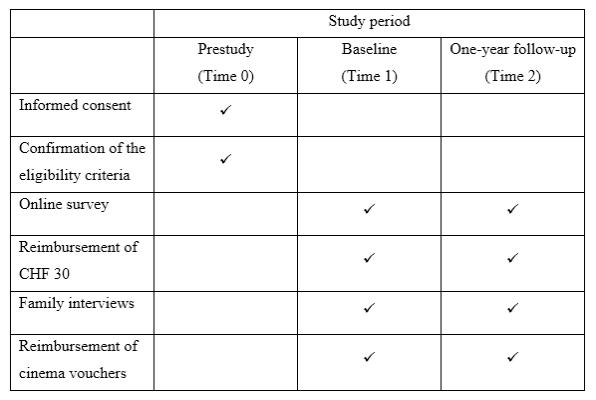
Overview of the study procedures.

The exclusion criteria are as follows: a Swiss parent, refugee status, foreign exchange student status, and insufficient comprehension of English to complete questionnaires or interviews. Parents who are interested in participating in the study will contact the study team by email/phone. We will then send the study information and informed consent documents by email/post. Following that, informed consent will be obtained either in person at the Division of Clinical Psychology and Psychotherapy or online via phone/Skype/Zoom with a member of the study team. Once they agree to participate in the study, parents and adolescents will have to sign the informed consent form. Children (aged 10-13 years) will have to write their names in bold on the informed consent form. For children below 10 years, oral consent will be obtained in the presence of a parent. Parents will have to return the signed informed consent forms to the study team via post or email. Once written informed consent has been received by the study team, we will proceed with the following study procedures. Participants will be reimbursed CHF 30 (US $30) upon completion of the online survey. Those who participate in the family interviews will be provided cinema vouchers (CHF 40 [US $40]). Similar procedures will be conducted at the 1-year follow-up. Figure 1 provides an overview of the study procedures.

### Quantitative Procedure

Each participant and one of their parents will receive a pseudoanonymous unique code in order to access the online survey. They will be asked to complete the online questionnaire that will last for about 10-15 minutes at their own convenience. Upon completion of the online questionnaire, parents will receive reports on their well-being and their child’s well-being if they have provided consent. For the parent, a score below 13 on the 5-item World Health Organization Well-Being Index (WHO-5) [46] indicates poor well-being and is an indication for further evaluation. For the child/adolescent, a score of 15 or above for Total Difficulties or a score of 1 or above for Impact Score on the Strengths and Difficulties Questionnaire (SDQ) [47] is an indication for further evaluation. In such cases, parents who have consented to receive this information will be contacted for a recommendation for further assessment at the Center for Psychotherapy, University of Basel. This initial evaluation will be conducted free of charge, but the cost of subsequent assessments/treatment will be borne by the participants. Participants and their families who have consented to be contacted for the 1-year follow-up study will be invited to complete the same online questionnaire again. They will need to provide informed consent again should they want to participate in the follow-up study.

### Measures

Quantitative data will be collected using an online questionnaire hosted on the university’s website. The following sociodemographic information will be collected: age, ethnicity, nationality, marital status, employment status, relationship with the child, spoken languages, number of previous international relocations, countries lived in before Switzerland, education, school type, and reasons and length of relocation. In addition, measures as listed in Textbox 1 will also be used.

**List of measures.**

**Predictor measures**

Psychological

- Emotion regulation: Emotion Regulation Questionnaire for Children and Adolescents [48]
- Negative self-thoughts: Social Threat Subscale of the Children’s Automatic Thoughts Scale [49]
- Psychological attributes: Strengths and Difficulties Questionnaire [47]
- Perceived stress: Perceived Stress Scale for Children [50]
- Resilience: Child and Youth Resilience Measure-12 [51]
- Self-esteem: Rosenberg Self-Esteem Scale [52] or Behavioral Rating Scale of Presented Self-Esteem in Young Children [53]

Cultural

- Acculturation stress: Acculturative Stress Inventory for Children [54]
- Cultural intelligence: Short-Form Cultural Intelligence Scale [55]

Family

- Family functioning: McMaster Family Assessment Device [56]
- Parent’s resilience: Adult Resilience Measure [51]
- Parental stress: Perceived Stress Scale [57]
- Quality of couple relationship: Couples Satisfaction Index-4 [58]

**Outcome measures**

Well-being

- KIDSCREEN-10 Index [59]
- 5-item World Health Organization Well-Being Index [46]

Sociocultural adjustment

- Social Cultural Adaptation Scale–Child [60]
- Social Cultural Adaptation Scale–Revised [61]

#### Outcome Measures

Our outcome measures include well-being and sociocultural adjustment.

##### Well-being

Third culture kids’ well-being will be assessed using KIDSCREEN-10 [59]. Children and adolescents will be asked to rate each item based on a Likert-type scale ranging from 1 (not at all) to 5 (extremely). Items are summed to provide a total score, with higher scores indicating higher levels of well-being. Parents’ well-being will be assessed using the WHO-5 [46]. Parents will be asked to rate each item based on a Likert-type scale ranging from 0 (at no time) to 5 (all of the time). Items are summed to provide a total score, with higher scores indicating higher levels of well-being.

##### Sociocultural Adjustment

Third culture kids’ sociocultural adjustment will be assessed using the 20-item child version of the Social Cultural Adaptation Scale–Child [60]. For children below 12 years old, parents will be asked to fill out the Social Cultural Adaptation Scale–Child on their behalf. Parents’ sociocultural adjustment will be assessed using the 11-item Social Cultural Adaptation Scale–Revised [61]. For both measures, items are rated based on a Likert-type scale ranging from 1 (no difficulty) to 5 (extreme difficulty). Items are summed to provide a total score. Higher scores indicate more difficulties in the new sociocultural environment, suggesting poorer adjustment.

#### Predictor Measures

Our predictor measures include psychological, cultural, and family factors.

##### Psychological Factors

Psychological factors will be evaluated using different assessments as presented below.

###### Emotion Regulation

Children and adolescents will be asked to complete the 10-item Emotion Regulation Questionnaire for Children and Adolescents [48] based on a 5-point Likert scale ranging from 1 (strongly disagree) to 5 (strongly agree). Items are summed to provide a total score, with higher scores indicating greater use of emotion regulation strategies.

###### Negative Self-thoughts

Children and adolescents will be asked to complete the 10-item Children’s Automatic Thoughts Scale [49]. Each item is rated on a 5-point Likert scale ranging from 0 (not at all) to 4 (all the time). Items are summed to provide a total score, with higher scores indicating a higher frequency of negative automatic thoughts related to social situations.

###### Resilience

Children and adolescents will be asked to complete the Child and Youth Resilience Measure-12 [51] based on a 3-point Likert scale ranging from 1 (no) to 3 (yes). Items are summed to provide a total score, with higher scores indicating higher levels of resilience.

###### Psychological Attributes

Third culture kids’ psychological positive and negative attributes will be assessed using the 25-item SDQ [47]. These 25 items are divided between the following 5 subscales: emotional symptoms (5 items), conduct problems (5 items), hyperactivity/inattention (5 items), peer relationship problems (5 items), and prosocial behavior (5 items). Parents will be asked to rate their child/adolescent based on a Likert-type scale ranging from 0 (not true) to 2 (certainly true). The total difficulties score is summed using the emotional symptoms, conduct problems, hyperactivity/inattention, and peer relationship problems subscales. Higher total scores indicate more difficulties. Higher scores on the prosocial behavior subscale indicate better prosocial behaviors.

###### Stress

Children and adolescents will be asked to complete the 13-item Perceived Stress Scale for Children [50] based on a Likert-type scale ranging from 0 (never) to 3 (a lot). Items 2, 5, 6, 9, 10, 12, and 13 are reverse scored, and all items are then summed to provide a total score. Higher scores indicate higher levels of stress.

###### Self-esteem

Adolescents will be asked to complete the 10-item Rosenberg Self-Esteem Scale [52] that measures global self-worth based on a 4-point Likert scale ranging from “strongly agree” to “strongly disagree.” Items 2, 5, 6, 8, and 9 are reverse scored, and all items are then summed to provide a total score. For children aged 12 years or below, parents will be asked to complete the 15-item Behavioral Rating Scale of Presented Self-Esteem in Young Children [53]. Parents will be asked to rate descriptions that best describe their child based on scores of 1 to 4. Items are summed, and a mean score is calculated. For both measures, higher scores indicate higher self-esteem.

#### Cultural Factors

Cultural factors will be evaluated using different assessments as presented below.

##### Acculturative Stress

Acculturative stress will be assessed using the 4-item Acculturative Stress Inventory for Children [54]. All participants will be asked to rate each item based on a Likert-type scale ranging from 0 (does not apply) to 5 (bothers me a lot). Items are summed to provide a total score, with higher scores indicating higher levels of acculturative stress.

##### Cultural Intelligence

Parents will be asked to complete the 10-item Short-Form Cultural Intelligence Scale [55] that assesses their experiences when interacting with people from other cultures. Each item is rated on a 5-point Likert scale ranging from 1 (not at all) to 5 (extremely well). Items are summed to provide a total score, with higher scores indicating higher levels of cultural intelligence.

#### Family Factors

Family factors will be evaluated using different assessments as presented below.

##### Family Functioning

Parents will be asked to complete the 12-item family functioning subscale of the McMaster Family Assessment Device [56] based on a Likert-type scale ranging from 1 (strongly agree) to 4 (strongly disagree). Odd items are reverse scored, and all items are summed to provide a total score. Higher scores indicate poorer family functioning.

##### Parental Stress

Parents will be asked to complete the 10-item Perceived Stress Scale [57] based on a Likert-type scale ranging from 0 (never) to 4 (very often). Items 4, 5, 7, and 8 are reverse scored, and all items are then summed to provide a total score. Higher scores indicate higher levels of stress.

##### Parental Resilience

Resilience in parents will be assessed using the Adult Resilience Measure [51]. Parents will be asked to rate each item on a Likert-type scale ranging from 1 (not at all) to 5 (a lot). Items are summed to provide a total score, with higher scores indicating higher levels of resilience.

#### Qualitative Procedure

A random sample of participants and their families from the quantitative study, who have given their consent to be contacted for the family interview, will be invited for a face-to-face interview. Participant recruitment will end when saturation occurs. Based on previous studies [62], we expect the sample size to be between 15 and 20 families. They will need to complete the informed consent again prior to the interview. Each family will be interviewed together for about 40-50 minutes. The interview will be conducted either at the Center for Psychotherapy or at venues where the participants are recruited. However, in view of COVID-19 pandemic measures, the family interview may be conducted online via Zoom. Subsequently, participants and their families, who have consented to be contacted again for the 1-year follow-up, will be invited for the interview again. They will need to provide informed consent again should they want to participate in the interview. Qualitative data consisting of family interviews will be video recorded. Questions related to their well-being, relocation experiences, cultural issues, and challenges and difficulties living in Switzerland will be asked. See Textbox 2 for a list of the interview questions.

**List of interview questions.**

**Questions for the entire family**

Experience:

1. What has been the experience of moving to Switzerland for...
  the family?
  each individual?
2. What have been the positive things about moving? What have been the negative things about moving? How has it changed the family?

Cultural issues:

1. Have you moved before?
2. If yes, what are the specifics about moving to Switzerland compared with your previous moves?
3. If no, how does Switzerland compare with your hometown?
4. What have you perceived about being in Switzerland, what do you notice about the Swiss culture, and what is your experience of locals?

Needs:

1. What advice would you give another family who is moving here?

**Questions for parents**

Challenges/difficulties:

1. How has it challenged the family? How did you cope with that?
2. How has your family changed since the move?
3. How do members of your family support each other? (how have you supported one another during this time?)
4. Is there anything I have not asked you about living in Switzerland that might be important for me to know?

**Questions for children**

1. How happy are you to live in Switzerland?
2. What has been easy/difficult? How has it changed the family?
3. What did you miss most?
4. Is there anything I have not asked you about living in Switzerland that might be important for me to know?

#### Ethics Approval

The study has been approved by the Ethics Committee within the Faculty of Psychology, University of Basel, Switzerland (study number 047-18-4). Written informed consent and assent in accordance with the Declaration of Helsinki will be obtained from participants and their parents prior to any study-related procedures. In order to ensure confidentiality, all data will be pseudocoded without personal identifiers. Participants may withdraw from the study at any time without any consequences.

#### Data Analysis

##### Research Questions 1 to 3

Research questions 1 to 3 involve the roles of emotion regulation, self-esteem, resilience, negative self-thoughts, cultural intelligence, acculturative stress, family functioning, parents’ stress, and couple satisfaction in changes to third culture kids’ well-being and sociocultural adjustment over time.

Based on a multiple linear regression model with 5 predictors, whereby the focus is on the increase in the variance accounted for by a specific predictor, our planned sample size would be sufficiently powered at 0.80 for a small to medium effect size based on Cohen *f*^2^ of 0.04-0.05, given α=.05 [63]. To answer research questions 1 to 3, multiple regression analyses will be conducted, with well-being or sociocultural adjustment at the 1-year follow-up as an outcome and the respective psychological, cultural, and family factors as predictors, thereby controlling for the baseline values of the respective outcome variables and for sociodemographics (eg, age, gender, family composition, number of previous international relocations, years lived in Switzerland, and school type). As dropouts are frequently encountered in longitudinal studies, we will use multiple imputation to deal with the problem of missing values. Multiple imputation is superior to traditional approaches, such as completer analysis, if missing values follow the “missing at random” pattern, which we consider a more realistic scenario than the “missing completely at random” pattern.

##### Research Question 4

Research question 4 involves a family’s perspective of the process of transitioning and adjusting to another country and culture.

Content analysis will be chosen to analyze the family interviews, as it is deemed suitable for mixed methods studies [64]. Data will first be transcribed by trained research assistants and imported to MAXQDA Analytics Pro (VERBI GmbH) for coding. A codebook will be developed to guide the coding process. Each transcribed interview will be coded independently by a study team member and a trained research assistant. Discrepancies will be discussed and resolved together by another study team member. Intercoder reliability will be calculated using the MAXQDA intercoder agreement function. Data will be later triangulated to examine the relationships between the study variables and to identify the similarities and differences in the findings from both qualitative and quantitative procedures [64].

## Results

Data collection started on August 25, 2019, and will end on July 1, 2022. As of March 15, 2022, a total of 138 children and 126 parents have completed the baseline online survey. In addition, 44 children and 48 parents have completed the 1-year follow-up online survey. A total of 8 families have completed the baseline family interview, while 4 families have completed the 1-year follow-up interview. Transcription of the interviews is on-going. Results from this study will be presented at various conferences and are expected to be published in peer-reviewed journals from late 2022 onwards.

## Discussion

### Expected Principal Findings

Our longitudinal study aims to examine the roles of psychological, cultural, and family factors for third culture kids’ well-being and sociocultural adjustment over time, and gain in-depth insights into the family’s perspective of the process of transitioning and adjustment in Switzerland. Specifically, we hypothesize that higher levels of emotion regulation, self-esteem, and resilience, and lower levels of negative self-thoughts and stress will significantly predict higher levels of well-being and adjustment among third culture kids. In addition, we hypothesize that higher levels of self-esteem, resilience, cultural intelligence, couple satisfaction, and family functioning, and lower levels of stress will significantly predict higher levels of well-being and adjustment among the parents of third culture kids. Therefore, understanding the relationships between these factors would provide a better understanding of the well-being and adjustment among the third culture kid population in Switzerland. Furthermore, by examining a wide variety of psychological, cultural, and family factors, we will gain insights into critical resources that help to promote well-being and adjustment among third culture kids and their families. For instance, given that third culture kids and their families are likely to experience significant periods of stress when they relocate, it is extremely important to consider how they manage this and what skills might be helpful in dealing with relocation and adjustment in a healthy way. The identification of difficulties in these specific areas would enable early intervention and prevention of more severe problems associated with relocation stress.

Other important aspects include knowledge and skills to deal with acculturative stress and boost cultural intelligence. However, evidence is mixed for the potential preventive effect of premove preparation programs, including cultural pretraining, as cross-cultural training has measurable effects on knowledge about the host country [65], but is not proven to be efficient for stress reduction or adaptation [66]. It is possible that previous approaches for supporting expatriates have not been as successful as expected, as they only worked on one of these areas and did not take into account the interactions between psychological, cultural, and family factors. In summary, findings from our study will provide a foundation for the development of an evidence-based multi-level intervention program addressing the various psychological, cultural, and family factors, which may help to promote positive well-being and adjustment among third culture kids and their families. Specifically, the program will provide support in developing psychological skills, improving family and couple functioning, and developing cultural intelligence in 3 levels. Level 1 will consist of a combination of cultural training and psychoeducation regarding adjustment, acculturation, stress, and common challenges of relocation. Level 2 will consist of a parent or child group program aimed at building skills in stress management, emotion regulation, resilience, managing negative cognitions, boosting self-esteem, and improving family functioning. Level 3 will consist of an individualized program based on the specific needs of the family.

### Strengths

The strengths of this study include the longitudinal design, as it addresses the scarcity of methodologically strong studies in this area [4]. The longitudinal approach is particularly appropriate for this area of research as it enables us to understand the progression of well-being and adjustment of third culture kids and their families over a period of 1 year. Another strength is that the use of a mixed methods concurrent triangulation design addresses the existing gap in the literature of limited studies that adopt both approaches for understanding third culture kids’ well-being and adjustment. In addition, adopting a family-focused and multi-informant approach enables us to gain a more holistic perspective.

### Limitations and Future Directions

The limitations include a lack of manipulation checks to detect the attention of study participants while completing the online survey. We assume that all participants will answer the online survey with full attention. To account for this issue, we will examine the response time of each participant in order to identify those who have answered the survey too quickly, and these outliers may be excluded. In addition, only one of the parents is being recruited for the online survey. It is possible that our findings may differ depending on which parent is recruited. Finally, our sample will only consist of third culture kids and their families living in Switzerland. Hence, findings from this study may have limited generalizability to third culture kids living in other countries.

With these limitations, more research is clearly needed to further understand the factors that may influence well-being and sociocultural adjustment among third culture kids and their families. First, future research should consider including each family member to provide a more holistic perspective of the relocation experience and adjustment. Second, it would be interesting to extend the research sample to those living in countries beyond Switzerland. Finally, additional factors beyond those examined in this study, such as coping styles, parenting, and self-care behaviors, should be considered.

### Conclusions

This study addresses an emerging issue of globalization. Relocation failure results in high costs for organizations, while unhappy and unsettled expatriate families may be at risk for psychosocial problems and may be a burden for other members of the community. Findings from our study may provide further understanding of the risk and protective factors associated with the well-being of third culture kids and their families, and could therefore be helpful in developing a tailored multi-modal and multi-level program to promote positive well-being and adjustment during their stay abroad.

## Abbreviations

SDQ: Strengths and Difficulties Questionnaire
WHO-5: 5-item World Health Organization Well-Being Index
